# Level of job satisfaction amongst nurses in the North-West Province, South Africa: Post occupational specific dispensation

**DOI:** 10.4102/curationis.v39i1.1438

**Published:** 2016-02-25

**Authors:** Sisinyana H. Khunou, Mashudu Davhana-Maselesele

**Affiliations:** 1 School of Nursing, Faculty of Agriculture Science and Technology, North-West University, Mafikeng Campus, South Africa

## Abstract

**Background:**

Job satisfaction and burnout have been recurring problems amongst nurses in the Republic of South Africa (RSA). As a result, nurses are still leaving the rural public sector in search of lucrative work at the urban and private sectors and in developed countries. Accordingly, Occupational Specific Dispensation (OSD) was introduced as a strategy to circumvent the problem. However, since the implementation of OSD in 2007, there have been no studies conducted regarding the level of job satisfaction amongst nurses after the implementation of OSD in the North-West Province, especially because each province has its own challenges that are unique to the area.

**Objectives:**

The study had two objectives: to describe the level of job satisfaction amongst professional and other category nurses (OCNs) at a public hospital in the North-West Province and describe the perceptions of nurses about OSD and their intention to continue working in the hospital.

**Method:**

A quantitative descriptive cross-sectional design was used. Stratified random sampling was used to select a sample of 92 professional nurses (PNs), 90 enrolled nurses and enrolled nursing assistants (*Nursing Act* 2005), which made a total of 182 participants. For the purpose of the study, the enrolled nurses and enrolled nursing assistants were referred to as OCNs. Data were collected using the Minnesota Satisfaction Questionnaire–short form and OSD statements and analysed with Statistical Package for Social Science (SPSS, version 18).

**Results:**

The majority of PNs (79.3%, *n* = 73) and OCNs (86.7%, *n* = 77) were dissatisfied with the working conditions and salary: PNs (80.4%, *n* = 74) and OCNs (87.8%, *n* = 79).The nurses mostly discredited the current state of the OSD implementation. Majority of the PNs (84%, *n* = 77) and OCNs (91%, *n* = 82) disagreed with the statement that ‘level of job satisfaction has improved after the implementation of OSD’.

**Conclusions:**

The National Department of Health should consider a holistic approach to address all work-related conditions for nurses in order to curb the attrition rates. Policy makers and unions should urgently review OSD for all categories of nurses.

## Background

Nurses play a pivotal role in determining the efficiency, effectiveness and sustainability of the healthcare system. As such, it is important to know and understand what variables satisfy and motivate them to continue working in public hospitals. Studies indicate that poor working conditions and organisational climates are strong predictors of nurses’ job dissatisfaction in the Republic of South Africa (RSA) public hospitals (Mokoka, Oosthuizen & Ehlers 2010:4; Pillay 2009a:59). In addition, studies conducted in other countries also highlight factors influencing nurses’ job satisfaction and retention (Alotaibi 2008:238; Coomber & Barriball 2007:29; Wilson *et al*. 2008:716). A study conducted in Zambia by Mulenga (2010:69) revealed that institutional, individual and local environmental factors influenced nurses’ retention in rural remote areas. In that regard, the importance of using a comprehensive approach when addressing retention problems was highlighted (Mulenga 2010:64). Another study done in Pakistan by Khaliq, Zia Ur-Rehman and Rashid (2011:985) identified human resource issues as the key factor contributing to nurses’ job satisfaction. According to Khaliq *et al*. (2011:985), managers need to identify work-related issues such as empowerment and fair treatment given that such identification of frustrations, turnover and stress can be addressed if contributing factors are known, hence the need for this study.

The consequences of nurses’ dissatisfaction are lack of retention, which also affects the healthcare delivery system, nurses and patients. In that regard, patient outcomes will be reduced, resulting in increased mortality rates (Oulton 2006:39). According to Oulton (2006:37), nurses will go where they are respected, rewarded for their problem-solving skills, challenged appropriately and given personal and professional development. The high shortage of nurses in the RSA is an enormous barrier to achieving the goals of the National Department of Health (NDoH), which are to make health care affordable, equitable and accessible to all (African National Congress 1994; United Nations Millennium Development Goals 2000).

There is a huge gap in salaries between professional nurses (PNs) in the RSA and other developed countries, thus influencing the emigration of the nurses to other countries (King & McInerney 2006:73; Oosthuizen & Ehlers 2007:20; Pillay 2009b:5). In addition, Egerdahl and Casale (2010:43) indicated that RSA’s PNs emigrating to the USA can expect to a salary that is 232% more. Nurses’ salaries were low compared to those of other health professionals and could not meet the demands of inflation (Egerdahl & Casale 2010:43). In support of this statement, Magana and Damons (2013:65) found that there was a correlation between salary and the well-being of nurses. The majority of the nurses agreed that remuneration and working conditions needed to be improved (Magana & Damons 2013:65).

According to the RSA NDoH (2011:30), certain push-and-pull factors affecting attrition and migration are pay, lack of education opportunities, rate of HIV and/or AIDS infections and poor working conditions. As a result, many RSA healthcare professionals work abroad. In the year 2011, the NDoH indicated that a total of 23 407 of the country’s healthcare professionals were working overseas: 3496 in Australia, 2360 in Canada, 1596 in New Zealand, 8999 in the UK and 6956 in the USA. Similarly, George and Reardon (2013:8) found that 59% of the nurses and doctors envisaged emigrating to overseas countries because of poor working conditions, pay and the risk of being infected with HIV and/or AIDS. George and Reardon (2013:8) also recommended that there should be a reassessment of the effectiveness of retention strategies and that the NDoH should improve the working conditions in the public health sector. A study done by Tshitangano (2013:8) found that nurses’ turnover is attributed to dissatisfaction with regard to staffing, salary, resources and workplace safety.

According to NDoH (2011:30), providing health services to rural communities is a challenge in the RSA whereby 43.6% of the population is served by 12% of doctors and 19% of nurses. In support of that, the NDoH (2011:31) indicated the vast difference that exists in the distribution of health care professionals amongst provinces in the RSA. The healthcare professionals per 10 000 in rural provinces were as follows: 45 in Limpopo and Mpumalanga Provinces and 33 in the North-West Province (NWP) (NDoH 2011:30). Similarly, Oulton (2006:38) highlighted that there were too many nurses in the cities and too few in rural areas that have a high demand for nurses. To address the shortage of nurses, hospital managers should create a positive practice environment (Oulton 2006:38).

In order to address issues of retention in the public sector, the RSA put strategies in place, which included implementation of Occupational Specific Dispensation (OSD) in accordance with the Public Health and Social Development Sectoral Bargaining Council (PHSDSBC). According to the PHSDSBC (2007:2), the objectives of this policy are as follows: to introduce the OSD remuneration and career progression system for PNs, enrolled nurses (ENs) and enrolled nursing assistants (ENAs) that provide for career pathing, pay progression, grade progression, recognition of appropriate experience, to have increased competencies and performance, to attract and retain nursing professionals in all identified occupations in the public health sector and to introduce differentiated salary scales for identified categories of nursing professionals based on a new remuneration structure.

Studies have been conducted by Ditlopo *et al*. (2013:141) and George, Atujuna and Gow (2013:8) with regard to the retention of nurses, but there is limited evidence of those specifically on nurses’ job satisfaction and OSD in the NWP. A study conducted in two provinces, namely, the NWP and the Free State by Ditlopo *et al*. (2013:141) indicated that there were several gaps with regard to the implementation of this policy. Insufficient attention was paid to time and resources, dependency relationships, task specifications, communication and coordination. According to Ditlopo *et al*. (2013:141), there were variations in the implementation of OSD policy in different provinces, facilities and settings. Therefore, OSD further contributed to more imbalances in the distribution of nurses between the hospital and Primary Health Care facilities as well as between the provinces.

Another study done in KwaZulu-Natal by George *et al*. (2013:8) found that OSD was effective in improving salaries of nurses, but there were other factors that impacted negatively on the motivation of healthcare workers. Healthcare workers’ decision to migrate was motivated by a mixture of factors including high levels of stress as a result of increased workloads and other challenges (George *et al*. 2013:8). In contrast, Kunene and Dolamo (2013) highlighted that the managers and speciality nurses indicated that OSD was effective in stopping migration, recognition of other post-basic qualifications, focus on career pathing, prevention of staff rotation and the general increase of salaries of all nurses. These studies did not investigate the level of job satisfaction amongst the nurses after implementation of OSD. Therefore, it was important to investigate the level of job satisfaction amongst nurses at a selected hospital in the NWP after the implementation of OSD.

## Problem statement

OSD was introduced in 2007 as a government strategy to attract and retain nurses in the public sector. However, anecdotal evidence mostly suggests that nurses’ job satisfaction remains low. Nurses are still migrating from the rural public sector to the private sector, urban provinces or even overseas. This has been attributed to several different push-and-pull factors. Failure to retain nurses in the public sector often leads to a reduction in patient outcomes, which in turn leads to increased mortality rates in predominantly rural provinces like NWP. The effectiveness of OSD in improving nurse job satisfaction and retention in rural public hospitals therefore remains questionable. Several studies were done with regard to the retention of the nurses, but there was limited evidence of those that were done specifically on the nurses’ job satisfaction and OSD in the NWP. The current healthcare challenges, including the growing HIV-related disease burden and all other noncommunicable diseases, stimulated interest to conduct the study on the level of job satisfaction and the perceptions about the success of OSD at the provincial level.

### Research questions

The following research questions were used to guide the study:

- What is the level of job satisfaction amongst professional and other category nurses (OCNs) at a public hospital in the NWP?
- What are the perceptions of nurses about OSD and their intention to continue working at the hospital?

### Purpose of the study

The purpose of this study was to describe the level of job satisfaction amongst nurses at a hospital in the NWP after the implementation of OSD.

### Objectives of the study

The specific objectives of the study were:

- to describe the level of job satisfaction amongst professional and OCNs at a public hospital in the NWP
- to describe the perceptions of nurses about OSD and their intention to continue working at the hospital in the NWP.

### Theoretical framework

The study followed Frederick Herzberg’s Two-factor theory of motivation as it is relevant to job enrichment (Daft 1999:244; Herzberg 1959). Herzberg’s theory states that satisfaction and dissatisfaction are driven by different factors − motivation and hygiene factors, respectively. The hygiene factors remove dissatisfaction and do not cause the employee to become highly satisfied and motivated (Daft 1999:244). Dissatisfying experiences called ‘hygiene’ factors result from extrinsic factors such as company policies, salary, co-worker relationships and supervisory style. The ‘motivation factors’ make the workers to be highly motivated and satisfied. If managers want to motivate their team, focus should be on factors like achievement, recognition and responsibility (Daft 1999:244; Herzberg 1959).

### Definition of terms

- *Job satisfaction* is defined as ‘one’s affective response to the job’ (Thompson & Phua 2012:276). In this study, nurses’ job satisfaction determines their intention to continue working in the public hospital.
- A *professional* nurse is one who has undergone training, acquired a nursing diploma or degree and is registered with the South African Nursing Council, as required according to *Nursing Act* Number 33 (2005:5). In this study, a nurse means a professional nurse working at public hospitals of the NWP.
- *EN* (enrolled nurses) means a person registered as such in terms of Section 31 of the *Nursing Act* Number 33 of 2005. This person is educated and competent to practise basic nursing in the manner and to the level prescribed (Nursing Act 2005:5). *ENs* is used for *OCNs.*
- *Enrolled nursing assistant* (ENA) is a person educated to provide elementary nursing care in the manner and to the level prescribed (Nursing Act 2005:5). *OCNs*: For this study, *ENs*. *ENAs* are referred to as OCNs.
- *OSD*: It is the agreement on remuneration and career progression system for registered nurses with a view of attracting and retaining nursing professionals in all identified occupations to the public health sector (PHSDSBC, Resolution No 3 (2007).

### Significance of the study

This study is important as the assumption is that: if the level of job satisfaction is known, policy makers can know whether nurses are satisfied in their jobs and appropriate measures can be taken to address those factors that cause dissatisfaction. The objective of the RSA government to retain nurses in public hospitals is dependent on such information. It is hoped that the findings will be useful for policy makers and planners, as well as hospital managers towards the development of conditions of service that will encourage nurses to remain in RSA hospitals, even in rural areas of the North-West Province. The findings of the study could also provide employers and trade unions best practice on how to improve work life, job satisfaction and staff retention.

## Research design and methods

### Design

A descriptive cross-sectional design (Brink, Van der Walt & Van Rensburg 2013:114) was used to describe the current level of job satisfaction amongst the different categories of nurses at a public hospital in the NWP. This approach was used to provide facts about the factors contributing to job satisfaction, job retention, intention to remain employed and their link to OSD at a public hospital in the NWP after the implementation of OSD. The study used the quantitative approach as it aimed to quantify its variables through the use of a questionnaire and afterwards compare its variables. According to Burns and Grove (2011:26), quantitative research is used to describe variables and determine the cause-and-effect interactions between the variables.

### Population and sampling

The population was made up of 335 nurses who received OSD and were working at a public hospital in the NWP. The population was chosen because they were all working at this hospital and could all have different views regarding the conditions that might contribute to job satisfaction or dissatisfaction. Probability-stratified random sampling was used to ensure that all categories of nurses had an equal chance of being included in the study (Polit & Beck 2004:295). The respondents were stratified according to their level of training, namely PNs, ENs, ENAs (*Nursing Act* 2005:5). For the purpose of this study, the ENs and ENAs were referred to as OCNs. The sampling frame was obtained from the monthly allocation to get the total number of nurses working at the hospital. All strata were represented proportionally, that is the stratum with more members had more representation.

### Sample size

According to Brink *et al*. (2013:131), a sample is a part or fraction of a whole, or a subject of a larger set selected by the researcher to participate in a research study. The sample size of at least 182 observations was determined using a sample size formula recommended by Sceaffer *et al*. (2006:46). The error rate in the calculation of sample size was set to 5 and 95% confidence intervals (Babbie *et al*. 2015). Of the 200 questionnaires distributed to increase the response rate, 182 (92 PNs and 90 OCNs) were returned (a response rate of 91%). The total sample was 182 (92 PNs and 90 OCNs).

### Data collection

Data collection is the precise, systematic gathering of information relevant to the research purpose or specific objectives and questions (Burns & Grove 2011:52). This section elaborates on the systematic methods of data collection to describe the level of job satisfaction amongst nurses at a hospital in the NWP after the implementation of OSD.

### Research instrument

Data were collected through the use of self-administered questionnaires. The questionnaire was made up of 37 questions that were divided into three sections, namely, demographic data, factors contributing to job satisfaction and dissatisfaction, and OSD statements. The instrument used was the Minnesota Satisfaction Questionnaire (MSQ) and the OSD statements that were obtained from literature and adapted so as to obtain data from the participants (Weiss *et al*. 1967).

The questionnaire was divided into three sections. Section 1 had nine items (one closed-ended and eight open-ended items).This section requested information about the nurses’ personal, educational and employment. Section 2 was made up of the MSQ 20 items classified according to the Herzberg‘s hygiene and motivation factors (Weiss *et al*. 1967).These items were closed-ended measures on a 5-point Likert scale ranging from very dissatisfied (1) to very satisfied (5). Section 3 contained eight items adapted from the OSD objectives (PHSDSBC 2007:4). The items were closed-ended questions measured with the use of the Likert scale, which ranged from 1 (strongly disagree) to 5 (strongly agree).

### Pilot study

The pilot study was conducted at local clinics different from the study site. A total of 30 nurses, of whom 10 represented each nursing category, were included in the pilot study. The sample was purposefully selected to ensure that all categories were included. The outcome of the pilot study offered information on the study feasibility, appropriateness and efficiency of the data collection process. Instruction on Section 1 was found to be irrelevant as participants were asked to ‘tick’ rather than ‘respond’ in the appropriate boxes. The item on experience as a nurse was also rectified as it only asked about PNs. These items were subsequently reconstructed to improve clarity. The respondents were able to answer the questionnaire within 20 minutes. In addition, the data from the pilot study were used to determine the reliability of the instrument.

## Validity and reliability

### Validity

Content validity is the assessment of how well the instrument represents all the components of the variable to be measured (Brink *et al*. 2013:160). The draft questionnaire was submitted to the supervisor for expert scrutiny regarding the relevance of each item. Pretesting of the questionnaire was done at three district clinics, which were not part of the study population. The researcher distributed 30 self-administered questionnaires to respondents to comment on the clarity of the questions.

### Reliability

According to Brink *et al*. (2013:164), three methods of reliability testing are referred to in the literature: stability, equivalence and internal consistency. In the study, internal consistency was ensured by: literature being reviewed to verify the internal consistency of the MSQ–short form. MSQ–short form was used to investigate nurses’ perceptions about their working conditions and their intention to stay in the regional hospital (Selebi & Minnaar 2007:30). The MSQ–short form with five subscales was used and found to have reflected a reliability of 0.80 Cronbach’s Alpha (Selebi & Minnaar 2007:30). A confirmatory analysis of reliability was performed on the pilot data using Cronbach’s Alpha. The Cronbach’s Alpha of the 20 items of the MSQ (0.878 which is greater than 0.7) showed that the scale is very reliable (Rose, Spinks & Canhoto 2014). In other words, 87.8% variation amongst the 20 items of MSQ was consistent across the items (Ruel, Wagner & Gillespie 2015). The Cronbach’s Alpha of the items of the OSD (0.902 which is much greater than 0.7) showed that the scale is highly reliable. That is, 90.2% variation amongst the eight items of OSD was consistent across the items.

### Data collection process

Data collection was done between 28 February and 30 April 2011. The researcher was personally responsible for the distribution and collection of all questionnaires. Questionnaires were distributed to the respondents in each ward and immediately collected for proper monitoring of the process. The data collection process was done during the day and at night to ensure adequate participation so as to allow participation of nurses who were on night duty at the time of the study.

### Data analysis

The SPSS, version 18 was used to analyse the generated data. Basic descriptive psychometric analysis of the MSQ instruments was done, which was followed by internal consistency assessment using the Cronbach’s Alpha (Cronbach 1951). Students’ *t*-test and analysis of variance were used to compare the various subscales by the demographic characteristics, including the level of education and training of the nurses (Brink *et al*. 2013:191). Regression analysis was used to identify the MSQ items contributing to job satisfaction (Brink *et al*. 2013:191). The chi-square statistical test was used to determine whether there was a relationship between the nursing category and the job satisfaction items. The 0.05 level of significance was used for this test. This test was also used to determine whether there was significant difference between the two categories of nurses (Burns & Grove 2011:401).

## Ethical considerations

In order to access nurses at the hospital in the NWP, ethical clearance was obtained from the North-West University‘s Ethics Committee (Mafikeng Campus). Permission to conduct the study was obtained from the North-West Provincial Health Department, the Chief Executive Officer and Deputy Director of Nursing of the public hospital wherein the study was conducted. The researcher also adhered to three fundamental ethical principles for protection of human rights throughout the study. Respect for persons was ensured by providing the nurses with adequate understandable information before signing the consent form. The participants were not coerced to participate in the study. The rights to privacy, anonymity and confidentiality were ensured by not writing the names of the participants on the questionnaires. Respondents were requested to sign an informed consent form when they agreed to participate in the study.

## Results

### Demographic characteristics

Table 1 indicates the basic demographic data of the respondents. This study had *n* = 182 respondents consisting of almost similar numbers of PNs (92) and OCNs (90). The results presented in this study are also separated and compared between these two categories of nurses. About 92% (*n* = 85) of the PNs and 96% (*n* = 86) of the OCNs were females. The majority (70%, *n* = 63) of the OCNs had Grade 12 and the remaining (30%, *n* = 27) had less than a grade 12 level of education. About (78.3%, *n* = 72) of the PNs had a college diploma compared to about 22% (*n* = 20) who had university degrees.

**TABLE 1:** Demographic and professional characteristics of the study participants (*n* = 182).

Characteristics	OCNs (*n* = 90)		PN (*n* = 92)	
	
	Frequency	Percentage	Frequency	Percentage
**Gender**				
Male	4	4.4	7	7.6
Female	86	95.6	85	92.4
**Marital status**				
Single	43	47.8	27	29.3
Married	33	36.7	52	56.5
Divorced	6	6.7	6	6.5
Widowed	8	8.9	7	7.6
**Level of education**				
Less than Grade 12	27	30.0	0	0
Grade 12	63	70.0	0	0
Diploma	0	0	72	78.3
Degree	0	0	20	21.7

OCNs, other category nurses; PN, professional nurses.

### Demographic and professional characteristics of professional nurses and other category nurses using the *t*-test

Table 2 shows the mean ages, number of children, years of experience as a nurse and years in a current unit for both category of nurses. The mean age of the OCNs was 43 (SD = 9.27) and that of the PNs was 44 (SD = 8.60). With regard to the years of experience as a nurse, OCNs had an average of 16.6 years (SD =10.92) and PNs had 19.14 (SD = 10.16) years. Both the PNs and OCNs had two children on average. The PNs had slightly more years on average (6.42) in the current unit compared to the OCNs (6.02) years. However, there was no significant difference in all four items compared as indicated in Table 2.

**TABLE 2:** Comparison of demographic and professional characteristics of PNs and OCNs using the analysis of variance (*n* = 182).

Characteristics	Nursing category group	N	Mean	SD	F	p
Age	OCNs	90	43.19	9.27	−0.760	0.448
	PNs	92	44.20	8.60		
Number of children	OCNs	90	2.08	1.13	−1.10	0.270
	PNs	92	2.26	1.10		
Years of experience as a nurse	OCNs	90	16.60	10.92	−1.62	0.106
	PNs	92	19.14	10.16		
Years working in current unit	OCNs	90	6.02	4.51	−1.79	0.075
	PNs	91	6.42	4.80		

OCNs, other category nurses; PNs; professional nurses.

### Level of job satisfaction amongst professional nurses and other category nurses

Table 3 shows participants’ description of the different items assessing their level of job satisfaction. The observed differences were mostly significant as shown by the *p*-values being less than 0.05. Out of the 20 items assessed, significant differences were found in 15 items. Table 3 also indicates that the highest proportion of dissatisfaction was noted with the items ‘my pay and the amount of the work I do’ and ’the working condition and environment’. In addition, majority of OCNs were dissatisfied with almost all the items as indicated in Table 3

**TABLE 3:** The level of job satisfaction amongst PNs and OCNs across the 20 items of the job satisfaction scale using the chi-square test (*n* = 182).

Job satisfaction assessment items	Nurse category (*n* = 182)	Dissatisfied *n* (%)	Undecided *n* (%)	Satisfied *n* (%)	*χ*^2^ (*p*)
My pay and the amount of work I do	PNs	74 (80.4)	11 (12)	7 (7.6)	3.120 (0.21)
	OCNs	79 (87.8)	9 (10)	18 (20.2)	
The working conditions and environment	PNs	73 (79.3)	8 (8.7)	11 (12)	8.952 (0.01)*
	OCNs	77 (86.7)	11 (12.2)	1 (1.1)	
The competence of my supervisor in making decisions	PNs	51 (55.4)	15 (16.3)	26 (28.3)	7.978 (0.02)*
	OCNs	56 (62.2)	23 (25.6)	11 (12.2)	
The way my boss handles his/her workers	PNs	50 (54.3)	21 (22.8)	21 (22.8)	1.132 (0.57)
	OCNs	54 (60)	21 (23.3)	15 (16.7)
The way company policies are put into practice	PNs	48 (52.2)	23 (25.0)	21 (22.8)	7.320 (0.03)*
	OCNs	50 (55.6)	33 (35.6)	8 (8.9)
The way my co-workers get along with each other	PNs	32 (34.8)	18 (19.6)	42 (45.7)	4.280 (0.12)
	OCNs	37 (41.1)	25 (27.8)	28 (31.1)
The chance to do something that makes use of my abilities	PNs	25 (27.2)	24 (26.1)	43 (46.7)	15.992 (0.00)**
	OCNs	47 (52.2)	24 (26.7)	19 (21.1)
The chance to try my own methods of doing the job	PNs	32 (34.8)	28 (30.4)	32 (34.8)	2.255 (0.32)
	OCNs	41 (45.6)	24 (26.7)	25 (27.8)	
The feeling of accomplishment I get from doing the job	PNs	(37 (40.2)	17 (18.5)	38 (41.3)	17.021 (0.00)**
	OCNs	47 (52)	30 (33.3)	13 (14.4)
The chance to be ‘somebody’ in the community	PNs	30 (32.6)	19 (21. 7)	42 (45.7)	8.257 (0.02)*
	OCNs	46 (51.1)	20 (22.2)	24 (26.7)
The praise I get for doing a good job	PNs	47 (51.1)	15 (16.3)	30 (32.6)	6.370 (0.04)*
	OCNs	59 (65.6)	15 (17)	15 (16.7)
The chance to work alone on the job	PNs	22 (23.9)	12 (13)	58 (63)	12.936 (0.00)**
	OCNs	41 (44.4)	17 (18.9)	33 (36.7)
The chance to do things for other people	PNs	18 (19.6)	12 (13)	62 (67.4)	21.195 (0.00)**
	OCNs	38 (42.2)	22 (24.4)	30 (33.3)
The chance to tell people what to do	PNs	26 (28.3)	10 (10.9)	56 (60.9)	14.590 (0.00)**
	OCNs	38 (42.2)	22 (24.4)	30 (33.3)
Being able to do things that do not go against my conscience	PN	29 (31.5)	20 (21.7)	43 (46.7)	10.713 (0.01)*
	OCNs	46 (51.1)	22 (24.4)	22 (24.4)
The chance to do different things from time to time	PN	39 (42.4)	18 (19.6)	34 (38)	15.344 (0.00)**
	OCNs	59 (65.6)	17 (21.1)	12 (13.3)
The chances for advancement on this job	PN	42 (45.7)	18 (19.6)	32 (34.8)	17.944 (0.00)**
	OCNs	65 (72.2)	16 (17.8)	9 (10)
The freedom to use my own judgement	PN	35 (38)	21 (22.8)	36 (39.1)	21.642 (0.00)**
	OCNs	57 (63.3)	24 (26.7)	9 (10)
The way my job provides for steady employment	PN	37 (40.2)	28 (30.4)	27 (29.3)	7.129 (0.03)*
	OCNs	54 (60)	18 (20)	18 (20)
Being able to keep busy all the time	PN	33 (37)	22 (23.9)	36 (39.1)	5.169 (0.08)
	OCNs	48 (53.3)	18 (20.0)	24 (26.7)

OCNs, other category nurses; PNs, professional nurses.

* Significant at *p* < 0.05;

** Significant at *p* < 0.01

The aggregate job satisfaction amongst PNs and OCNs were based on the subscales and total job satisfaction scores using the *t*-test.

Table 4 indicates the comparison between the aggregate scores of the PNs and OCNs on the 10 subscales of the job satisfaction questionnaire and the aggregate score for the entire questionnaire. The responses to the various questions ranged from dissatisfied to satisfied and with scores ranging from one (1) to three (3). The lowest score (1) was allocated to dissatisfied whilst the highest score (3) was allocated to satisfied. The aggregate score for each subscale was derived from the average score of the items that constitute the particular subscale. The total satisfaction score was a sum of all the 20 items measuring job satisfaction and ranged from 20 to 60. For all the subscales and job satisfaction score, PNs scored higher than the OCNs.

**TABLE 4:** The aggregate job satisfaction amongst PNs and OCNs based on the subscales and total job satisfaction scores using the *t*-test (*n* = 182).

Subscales and total job satisfaction score	Nursing category (*n* = 182)	N	Mean	SD	T	p
Salary	OCNs	90	1.14	0.41	−1.67	0.09
	PNs	92	1.27	0.59		
Working condition	OCNs	90	1.14	0.38	−2.21	0.02*
	PNs	92	1.32	0.68		
Supervision	OCNs	90	1.53	0.64	−1.61	0.10
	PNs	92	1.70	0.79		
Policies	OCNs	90	1.53	0.65	−1.57	0.11
	PNs	92	1.70	0.81		
Interpersonal relations	OCNs	90	1.90	0.84	−1.61	0.10
	PNs	92	2.10	0.89		
Achievement	OCNs	90	1.71	0.67	−3.39	0.00**
	PNs	92	2.06	0.74		
Recognition	OCNs	90	1.63	0.68	−3.09	0.00**
	PNs	92	1.97	0.79		
Responsibility	OCNs	90	1.86	0.74	−4.35	0.00**
	PNs	92	2.33	0.70		
Advancement	OCNs	90	1.44	0.56	−5.28	0.00**
	PNs	92	1.95	0.72		
Work itself	OCNs	90	1.66	0.73	−2.66	0.01*
	PNs	92	1.95	0.73		
Job satisfaction total score	OCNs	90	32.32	8.54	−5.01	0.00**
	PNs	92	39.09	9.62		

OCNs, other category nurses; PNs, professional nurses.

* Significant at *p* < 0.05;

** Significant at *p* < 0.01

### Perceptions of nurses about occupational specific dispensation and their intention to continue working in the hospital

Table 5 shows the perceptions of PNs and OCNs about the OSD and their intention to continue working in the public service. The majority of the respondents disagreed with statements about the current success of the OSD. There was no significant difference between proportion of PNs and OCNs who responded to the following statements: ‘Nurses are retained in the hospital since OSD implementation’ (*p* = 0.11), ‘level of job satisfaction has improved after implementation of OSD’ (*p* = 0.11), and ‘intention to keep working in the hospital for the next five years’ (*p* = 0.98).

**TABLE 5:** Perceptions of PNs and OCNs about OSD using the chi-square test (*n* = 182).

Perceptions about OSD	Nurse category (*n* = 182)	Disagree *n* (%)	Undecided *n* (%)	Agree *n* (%)	*χ*^2^ (*p*)
Satisfied about pay progression implemented on my salary	PNs	74 (80.4)	6 (6.5)	12 (13)	7.532 (0.02)*
	OCNs	92 (91.1)	6 (6.7)	2 (2.2)	
My experience has been considered in OSD implementation	PNs	67 (72.8)	7 (7.6)	18 (19.6)	19.687 (0.00)**
	OCNs	83 (92.2)	7 (7.8)	0	
OSD improved opportunities for development	PNs	53 (57.6)	20 (21.7)	19 (20.7)	18.366 (0.00)**
	OCNs	77 (85.6)	9 (10)	4 (4.4)	
OSD improved my performance	PNs	62 (67.4)	14 (15.2)	16 (17.4)	21.355 (0.00)**
	OCNs	83 (92.2)	7 (7.8)	0	
OSD attracted nurses to the hospital	PNs	73 (79.3)	6 (6.5)	13 (14.1)	14.300 (0.00)**
	OCNs	80 (88.9)	10 (11.1)		
Nurses are retained in the hospital since OSD implementation	PNs	73 (79.3)	7 (7.6)	12 (13)	4.335 (0.11)
	OCNs	77 (85.6)	9 (10)	4 (4.4)	
You intend to remain in the hospital in the next five years	PNs	59 (64.1)	23 (25)	10 (10.9)	0.039 (0.98)
	OCNs	58 (64.4)	23 (25.6)	9 (10)	
Level of job satisfaction has improved after implementation of OSD	PNs	77 (83.7)	6 (6.5)	9 (9.8)	4.507 (0.11)
	OCNs	82 (91)	6 (6.7)	2 (2.2)	

OCNs, other category nurses; PNs, professional nurses.

* Significant at *p* = 0.05;

** Significant at *p* = 0.01

## Discussion of results

### Demographic characteristics

The study revealed that 92% of the PNs and 96% of the OCNs were female. Similarly, studies found that the majority of respondents were female (Selebi & Minnaar 2007:30; Van der Westhuizen, Matheus & Reddy 2008:23). This was because of the fact that nursing has been predominantly a female profession that previously failed to attract males (Klaas, Mavundla & Yako 2007:47; Selebi & Minnaar 2007:3). The study found that the PNs had more experience compared to the OCNs. Consistently, Nkomeje and Nkosi (2008:75) revealed that the length of employment can influence nurses’ perceptions of the adequacy of promotion and remuneration. According to Mdindela and Werner (2009:109), employees who are employed more than 5 years is an indication of a relatively stable work force.

### Level of job satisfaction amongst professional nurses and other category nurses

The PNs and OCNs were compared on job satisfaction items on the MSQ using the Student’s *t*-test. The testing was done at the 0.05 level of significance. Several hygiene factors and motivation factors were strongly associated with the nurses being dissatisfied with their job. These factors were congruent with Herzberg‘s theory (Daft 1999:244). The hygiene factors that contributed to dissatisfaction were, low salary, poor working conditions, the way policies were implemented and the incompetency of the supervisor. The motivation factors that contributed to nurses’ job satisfaction were achievement, advancement, responsibility, work itself and recognition.

The study found that there was no significant difference (*p* = 0.21) regarding salary as perceived by both PNs and OCNs. As a result, PNs (80.4%, *n* = 74) and OCNs (87.8%, *n* = 79) were dissatisfied with salary, which meant that their needs could not be met by their salaries. In support, George and Reardon (2013:8) indicated that few participants perceived salary as an important push factor. According to Ngwenya and Makhubela-Nkondo (2009:485) and Pillay (2009a:52), nurses’ remuneration in the public sector is an enduring one and could be related to the move of many public sector nurses to the private sector. Pillay (2009a:52) also recommended that hospital managers should consider nonfinancial psychological rewards such as career opportunities, support and appreciation.

The study revealed that PNs (79.3%, *n* = 73) and OCNs (86.7%, *n* = 77) were dissatisfied with the working conditions and environment. Factors related to job satisfaction in this domain included a clean environment, adequate equipment and supplies (Mohale & Mulaudzi 2008:62). Poor conditions in the workplace influence nurses’ intentions to leave their organisations (George & Reardon 2013:5; Kruse & Van Dyk 2011:45; Mokoka, Oosthuizen & Ehlers 2010:6; Sojane *et al*. 2012:98). Similarly, George and Reardon (2013:8) found that doctors and nurses perceived emigrating overseas because of poor working conditions. In addition, Hlabahlaba and Seekoe (2014:93) found that there was a shortage of nurses in Eastern Cape healthcare centres, which was attributed to lack of safety. This resulted in long queues and verbal abuse by patients towards the nurses, which further exacerbated dissatisfaction. Seemingly, the nurses’ working conditions did not improve after the implementation of OSD. The findings of this study are congruent with Herzberg’s theory.

The present study found that 62% (*n* = 56) of OCNs and 55% (*n* = 51) of the PNs were dissatisfied with the competence of their supervisors in making decisions. In support, Hennessy and Minnaar (2009:76) revealed that management was inconsistent, unappreciative and generally unsupportive. According to Herzberg‘s theory, supervisors should apply appropriate managerial strategies (Herzberg 1959). Mokoka *et al*. (2010:8) reported that good managerial skills promote nurses’ job satisfaction and retention. Important managerial skills include respect and consistent feedback about performance.

It was revealed in the study that OCNs (56%, *n* = 50) and PNs (52%, *n* = 48) were dissatisfied with the way policies were put into practice. The findings support Herzberg’s theory, which stipulates that administration policies form an important role in employee dissatisfaction. Similarly, Nkomeje and Nkosi (2008:82) indicated that nurses need to be informed about hospital rules and procedures. The promotion of a positive work environment includes the involvement of nurses in decision-making processes (Nkomeje & Nkosi 2008:82).

The present study indicated that the majority of OCNs at 52% (*n* = 48) were dissatisfied with the subitems on achievement as compared to 27% (*n* = 24) of the PNs. The OCNs were not able to work independently and apply their knowledge and skills and thus not eligible for promotion and advancement. In addition, majority (72%, *n* = 65) of the OCNs when compared to the PNs (46%, *n* = 42) were dissatisfied with the career advancement factor. Similarly, Van der Westhuizen *et al*. (2008:43) implicated limited career advancement as the most relevant reason contributing to the participants leaving the public sector. This statement supports Herzberg’s theory, which states that the amount of achievement and recognition that an employee perceives are directly related to the level of job satisfaction (Daft 1999:244).

The highest proportion of PNs reported being satisfied with ‘the chance to do things for other people’ (67%, *n* = 62), ‘the chance to work alone on the job’ (63%, *n* = 58) and ‘the chance to tell people what I do’ (61%, *n* = 56). The satisfaction of the OCNs with regard to the same items were ‘the chance to do things for other people’ (33%, *n* = 30), ‘the chance to work alone on the job’ (38%, *n* = 33) and ‘the chance to tell people what I do’ (33%, *n* = 30) (Table 3). The observed differences were mostly significant as shown by *p*-values less than 0.05. Dissatisfaction amongst OCNs could be attributed to the fact that they did not benefit from OSD according to the PHSDSBC (2007:4). The findings are congruent with Herzberg’s theory. The supervisory role of PNs can also be related to their intrinsic motivation and increased job satisfaction. According to Selebi and Minnaar (2007:39), the dissatisfaction amongst lower category nurses could be attributed to the fact that their competence does not allow them to delegate others. Similarly, Sojane *et al*. (2012:103) recommended that all supervisors should praise and recognise their subordinates.

### Perceptions of nurses about occupational specific dispensation and their intention to remain working in the hospital

The majority of the nurses disagreed with all items about OSD. As given in Table 4, PNs (79%, *n* = 73) and OCNs (89%, *n* = 80) disagreed that OSD attracted nurses to the hospital. In addition, 79% (*n* = 73) of the PNs and 86% (*n* = 77) of the OCNs disagreed that nurses are retained in the hospital since the implementation of OSD. Both the PNs (64%, *n* = 59) and the OCNs (64%, *n* = 58) indicated that they do not intend to remain in the hospital in the next 5 years. It can therefore be concluded that the OSD objectives were not met, because the main objective of this policy was to attract and retain the nurses (PHSDSBC 2007:2). The dissatisfaction could be attributed to other hygiene factors such as the working conditions and supervision. The study is consistent with Herzberg’s theory of motivation, which stipulates that both factors should be considered to motivate employees and retain them. Seemingly, OSD did not address the hygiene factors and motivation factors as stipulated by the Herzberg’s theory. Even if nurses are given OSD, they should be provided with a conducive environment.

The PNs and OCNs mostly discredited the current state of OSD implementation. The study found that OCNs (56%, *n* = 50) and PNs (44%, *n* = 40) disagreed with all statements on OSD. In addition, the study revealed that there was no significant difference between the PNs and OCNs disagreeing with the statement on ‘level of job satisfaction has improved after the implementation of OSD’ (*p* = 0.11). The majority of the PNs (84%, *n* = 77) and OCNs (91%, *n* = 82) disagreed with this statement. This could be related to the fact that the nurses were dissatisfied with the implementation of OSD. The findings are congruent with those found by Motsosi and Rispel (2012:144), who indicated that OSD implementation was not done properly because of misinterpretation of some clauses in the policy. In support of these findings, Ngwenya and Makhubela-Nkondo (2009:482) found that workshops were not conducted by the DoH before the implementation of the OSD. The OSD policy was interpreted differently, which led to confusion with regard to implementation.

Seemingly, OSD did not make a difference on nurses’ salary. This is also supported by Tshitangano (2013:4), who indicated that the Limpopo Province nurses were dissatisfied with their salaries. According to Ditlopo *et al*. (2013:19), there were several weaknesses and unmet preconditions for OSD policy implementation. Insufficient attention was paid to time and resources, task specification, and coordination. Lack of compliance was because of unaccountability as no one took responsibility. This resulted in a vicious cycle of blame between different stakeholders including the South African Nursing Council (Ditlopo *et al*. 2013:19). It is clear that managers and unions should review OSD policy and identify barriers to its implementation.

## Limitations of the study

The findings of the study are limited to one public hospital in one district in the NWP and are thus not meant to be generalized to all districts, public hospitals and clinics in the NWP. It is suggested that the same study be conducted in the broader setting to determine the level of nurses’ job satisfaction after the implementation of OSD.

## Recommendations

- Nurses’ job satisfaction is complex and is influenced by both hygiene and motivation factors. Therefore, holistic comprehensive strategies including research, education, policies and nursing practice need to be put in place to address the nurses’ job satisfaction. The following recommendations are proposed based on the findings of the study: Nurses who take care of patients with HIV and/or AIDS and TB should also be remunerated and recognised as specialists as they are likely to be emotionally drained, overworked and at risk of being infected. This will serve as a means of appreciation and thus motivation.
- Policy makers and unions should urgently review OSD and other incentives for all categories of nurses and ensure the alignment of nurses’ remuneration with other health professionals. Hopefully, this will assist to ensure proper remuneration of all nursing categories and improve job satisfaction.
- The NDoH needs to invest in the future and mentoring of young nurses, because when nurses are exposed to a learning environment and development, they are likely to be stimulated to aspire to higher learning, which will further increase intrinsic motivation.
- Unit managers should be actively involved in strategic planning on human resource issues, for example, making inputs with regard to the number of nurses required for specific units by looking at the types of patients nursed in particular units.
- Hospital managers should also create opportunities for promotion in situations where staff advancement is identified as a problem. This means that the management of the hospital should attend to staff promotions to increase the intrinsic job satisfaction of their nursing staff.
- Qualitative research should be conducted to get an in-depth information about factors influencing job satisfaction in a provincial hospital.
- Impact of nurses’ working conditions on patient satisfaction should be researched to take corrective measures based on scientific research.

## Conclusion

In conclusion, the nurses’ level of job satisfaction remains low after the implementation of OSD. Both PNs and OCNs are dissatisfied with the hygiene factors. The nurses were highly dissatisfied about the working conditions and salary. The OCNs were dissatisfied with the hygiene and motivating factors. The study agreed with Herzberg’s theory of motivation. Therefore, based on the above results, the NDoH should address the nurses’ job satisfaction holistically.
